# Flexible Green Supply Chain Management in Emerging Economies: A Systematic Literature Review

**DOI:** 10.1007/s40171-022-00321-0

**Published:** 2022-11-04

**Authors:** M. K. Dhillon, P. M. Rafi-Ul-Shan, H. Amar, F. Sher, S. Ahmed

**Affiliations:** 1 University of Westminster, London, NW1 5LS UK; 2 University for the Creative Arts, Epsom, KT18 5BE UK; 3 Cardiff Metropolitan University, Cardiff, CF5 2YB UK; 4 Nottingham Trent University, Nottingham, NG1 4FQ UK; 5 University of West of Scotland, Paisley, PA1 2BE UK; 6 King Fahd University of Petroleum and Minerals, Dhahran, Saudi Arabia

**Keywords:** Drivers and barriers, Emerging economies, Flexible supply chain management, Green supply chain management, Strategies and practices, Systematic literature review

## Abstract

The purpose of this research is to synthesize the fragmented extant knowledge on flexible and green supply chain management (FGSCM) in the context of emerging economies and to unearth research gaps to motivate future research. We adopted a novel structured systematic literature review by triangulating a systematic literature review, text mining, and network analysis. Institutional theory and contingency theory were employed to analyze the results of the review. The results show that, firstly, research on FGSCM in emerging economies, despite its importance, is immature compared to general FGSCM literature. Second, the specificities of strategies and practices that distinguish this topic in emerging economies are discussed and the drivers and barriers are identified with respect to sources of institutional pressure. Third, a research framework for FGSCM in emerging economies is developed and 12 gaps for future research are identified. This study has exclusively developed a research framework for FGSCM in an emerging economy which has received the least consideration in the literature and practice. The framework was developed to synthesize the existing literature and to identify the research gaps to inspire future research.

## Introduction

Economic growth and consumerism have placed greater demands for energy and material consumption, resulting in increased concerns for environmental and natural resource preservation (Jia et al., 2018). Environmental issues are prime concerns for global economies due to global warming, increased pollution and depleting non-renewable resources (Malviya & Kant, 2015). Supply chain advancements since the 1990s introduced a new perspective that integrates environmental management within business operations to achieve a competitive advantage (Srivastava, 2007). Moreover, dissemination of flexibility from the manufacturing sector to the interorganizational and supply chain level created exciting, yet under-researched, opportunities for supply chain flexibility (Singh et al., 2020a, 2020b; Stevenson & Spring, 2007; Wadhwa & Rao, 2004).

Globalization resulted in increased demand for various products in the mid-twentieth century forcing global organizations to enter into new contexts of production where they had never operated before (Rajeev et al., 2017). The geographic extension of the supply chain resulted in more than 20% of the greenhouse gas to be emitted by organizations venturing on global platforms, due to the increased complexity of the sourcing and distribution channels as well as the socio-economic conditions of different countries (Dubey et al., 2017). Globalization not only created concerns vis-à-vis the ecological impact of supply chains, but also affected recent supply chain disruptions due to the Covid-19 pandemic. From this, it was established that the supply chain must be built flexibly to survive in volatile environments (Butt, 2021; Stevenson & Spring, 2007).

Growing environmental concerns and stringent environmental laws in developed countries have driven global companies to outsource the most polluting segments of their businesses to developing nations (Dwivedi et al., 2021; Garcia-Saravia Ortiz-de-Montellano & van der Meer, 2022; Geng et al., 2017a, 2017b). Among developing countries, emerging economies (e.g., Brazil, Russia, India, China, and South Africa, known as BRICS) are prime targets of multinational corporations due to a large consumer base, relatively disposable income, rapid industrialization, diversity of supply base, availability of skilled workforce, and lower operating costs (Tumpa et al., 2019). Emerging economies welcome foreign direct investment and benefit directly from globalization. This shift of location, however, has increased environmental concerns in emerging nations as well as the need for stricter environmental and social standards (Geng et al., 2017a, 2017b). On the other side, focal supply chain firms understood that the shift to emerging economies, despite the lower operating costs and new markets, involves unforeseen risks, which are specific to these countries. These risks can be mitigated only if the supply chain is proactive and if flexibility is built in supply chains in advance (Settembre-Blundo et al., 2021; Tukamuhabwa et al., 2017).

Recently, supply chain flexibility is becoming an attractive area of research for researchers and academicians (Singh et al., 2020a, 2020b). There is some research in this area, such as by Singh et al., (2020a, 2020b), who focus on measuring the performance of supply chain flexibility of an Indian soap manufacturing firm. Another research by Singh et al., (2020a, 2020b) focuses on mapping the causal relations among various supply chain flexibility dimensions and their impact on the Indian hygiene industry. However, there is limited research in the context of flexible green supply chain management (FGSCM). Research on FGSCM in emerging economies has been limited due to the contextual specificities of these countries such as the uncertainty inherent in their business environment and poor infrastructure to deal with sustainability issues (Silvestre, 2015; Singh et al., 2020a, 2020b). For example, 90% waste in India is dumped in the environment due to the lack of waste treatment and disposal facilities (Soda et al., 2015). Western corporates widely source from these manufacturers and service providers due to the availability of cheap labor and material. However, they have limited understanding of the context and poor visibility over the operational practices of their supply chains in such emerging economies (Rosin et al., 2020; Singh et al., 2019, 2020a, 2020b). Consequently, they are subject to scandals such as the Rana Plaza disaster in Bangladesh and enduring criticisms of overlooking environmental issues in their supply chain operations carried out in emerging economies (Bin Makhashen et al., 2020). Moreover, supply chain environmental issues exacerbated by disruptions in the recent pandemic have shown that sustainability and flexibility should be considered jointly in supply chain management, a need that has not been addressed in the literature hitherto (Paul & Chowdhury, 2020; Sassanelli & Terzi, 2022; Shibin et al., 2016).

Our initial review of the literature on FGSCM identifies the following gaps:

- Limited research has been conducted on FGSCM in the context of emerging economies, despite the increasing trend of operations and procurement from these countries (Singh et al., 2019, 2020a, 2020b)
- Despite the interconnectedness of flexibility and environmental sustainability in the supply chain management context, the literature has investigated these topics separately.
- Existing frameworks for flexible *or* green supply chain management fall short of utility for emerging economies due to inherently different characteristics of the business environment in these countries.
- While some systematic literature reviews in emerging economies have been conducted on flexible *or* green supply chain management barriers (Rahman et al., 2019; Shibin et al., 2016; Tumpa et al., 2019) and organizational performance (Geng et al., 2017a, 2017b), the literature scants systematic reviews that unearth the *specificities* and the *sources of institutional pressures* in emerging economies.

This review seeks to bridge these gaps by conducting a systematic literature review on FGSCM in emerging economies and addressing the following interrelated research questions -

i. What is the status quo of FGSCM research in emerging economies?
ii. What are the specificities of FGSCM strategies and practices in emerging economies?
iii. What are the sources of institutional pressure, i.e., drivers and barriers, to adopt FGSCM strategies and practices in emerging economies?
iv. How can the extant body of knowledge inform future research on FGSCM in emerging economies?

This article responds to the calls for further work on the FGSCM in emerging economies by proposing an innovative methodology that triangulates data from eclectic methods through a systematic literature review, text mining, and network analysis supported by two organizational theories for the cross-validation of findings and to eliminate subjectivity from the selection and review process. Three contributions to the literature of supply chain management are made. First, this article brings together the interrelated, yet separate, research developments in flexibility and environmental sustainability in supply chain management in emerging economies within the past 20 years. Second, it juxtaposes the FGSCM strategies and practices in emerging economies with the ones from the general literature, and thus unearths the contextual specificities of emerging economies using a contingency theory lens. Third, it identifies the sources of pressures that motivate or hinder FGSCM in emerging economies using institution theory to help policy makers advocate for FGSCM drivers and tackle the barriers.

The rest of this article is organized as follows. Section 2 presents the research design, theoretical perspectives, and the methodology used for the systematic literature review. Section 3 presents the results of a systematic literature review and provides infographics of the important trends found in the literature. Section 4 provides a thematic analysis of the results by identifying the strategies, practices, barriers, and enablers of FGSCM in emerging economies and comparing them against general FGSCM literature using the theoretical perspectives. Section 5 unearths the research gaps for future research and develops a research framework for FGSCM in emerging economies. Finally, Sect. 6 concludes the paper and provides the limitations.

## Research Design and Methodology

**Fig. 1:**
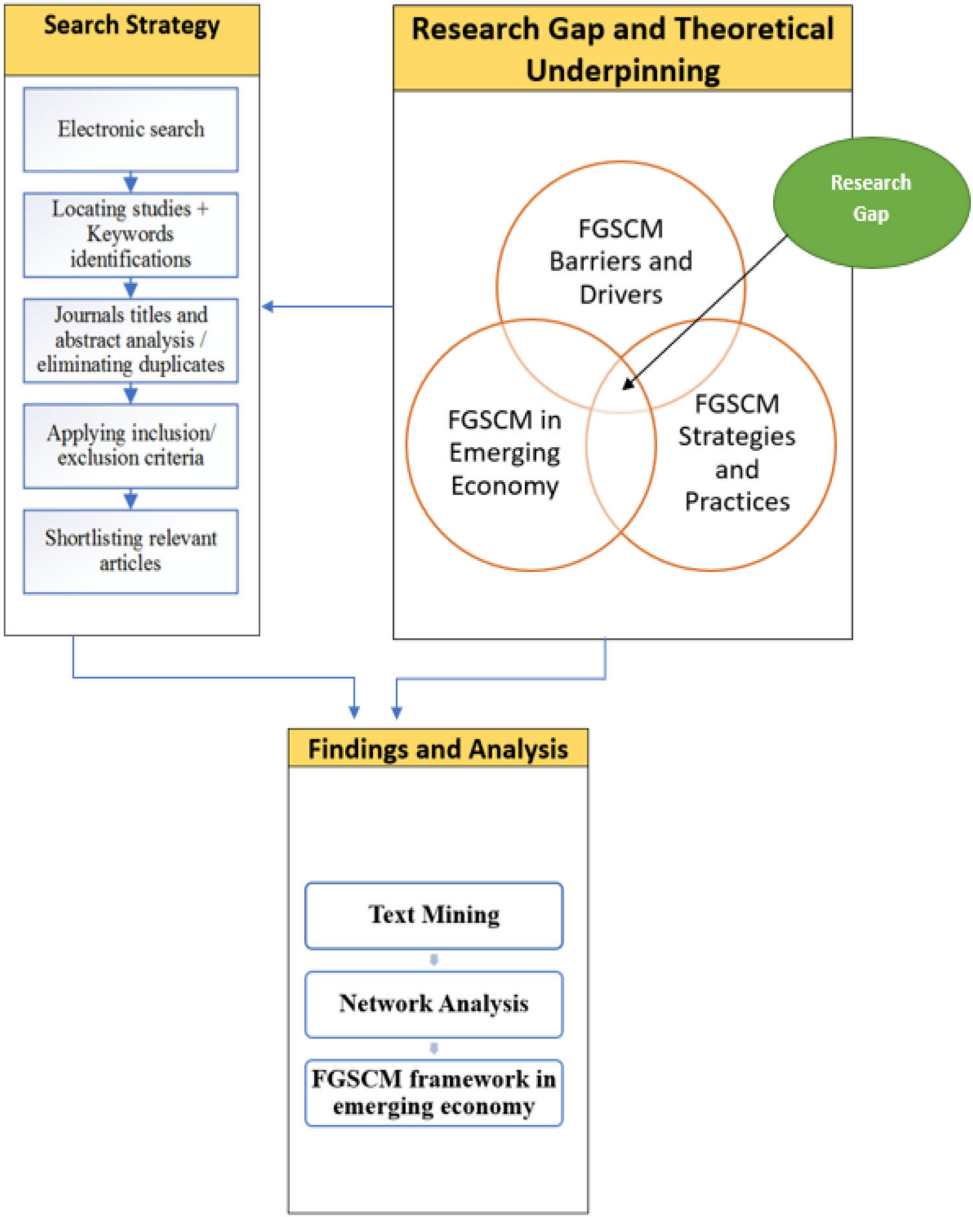
The research design and methodology for the systematic literature review

This section describes the research design, including the methodology, theoretical underpinning, and analysis approaches. The research gap, as discussed in Sect. 1, is where flexible SCM, green SCM, and SCM in emerging economies overlap. Motivated by this research gap, our proposed methodology triangulates different approaches to extract, analyze, and synthesize extant literature on FGSCM in emerging economies. It combines a systematic literature review, text mining, and network analysis to identify, evaluate, and synthesize the existing research (Denyer & Tranfield, 2009). An eclectic theoretical underpinning of contingency theory and institutional theory is adopted throughout the review. The methodology integrates the findings to build a theoretical framework for FGSCM in emerging economies. Figure 1 shows the research gap, theoretical lens, and the steps of the proposed methodology.

### Theoretical Perspective

A combined contingency theory and institutional theory lens was used to interpret the selected articles and develop a research framework. Contingency theory (Lawrence & Lorsch, 1967) is a major theoretical lens that expresses different organizational systems are the results of differences in their operating context. Since one of the objectives of this research is to understand the specificities of FGSCM in emerging economies, it can serve as an appropriate theoretical lens to scrutinize the differences between FGSCM strategies and practices in developed and emerging economies. Furthermore, we considered drivers and barriers as sources of positive and negative pressures, respectively, on the firms to adopt FGSCM strategies and practices. We take an institutional theory perspective (DiMaggio & Powell, 1983) to identify the source of drivers and barriers in emerging economies and discern whether they emerged to comply with regulations (coercive), to copy competitors or cope with cultural cognitive pressures (mimetic), or if they are in response to customer pressure (normative).

### Stages of the Methodology

The methodology started with a search in electronic databases to locate, select, and evaluate extant studies. First, relevant keywords were identified based on the internal discussion of authors, all of whom are academics with a background in supply chain and operations management. A corporate practitioner from India, experienced in FGSCM, was involved in the discussions at a later stage to ensure the viability of the keywords. The initial keywords were refined into series of search strings using Boolean logic, for example, "Green AND/OR Supply Chain," and "Emerging AND/OR Economy AND/OR Flexible AND/OR Supply Chain." Nearly synonymous keywords such as "Developing Country" or "Sustainable/Ethical Supply Chain" were also used. The search strings were continuously refined, resulting in 26 of the most relevant strings that were used to search data on *Web of Science*, *Science Direct*, *ABI/INFORM* and *Emerald Insight*. The following exclusion criteria, as proposed by Newbert (2007), were used to narrow the results down to those which were more relevant.

- Articles should be published in peer-reviewed scientific journals in English.
- Only journals in the area of logistics, operations management, and supply chain management are included.
- Articles should be published in the last 20 years.
- Articles must contain at least one of the keywords in their title or abstract.

**Fig. 2:**
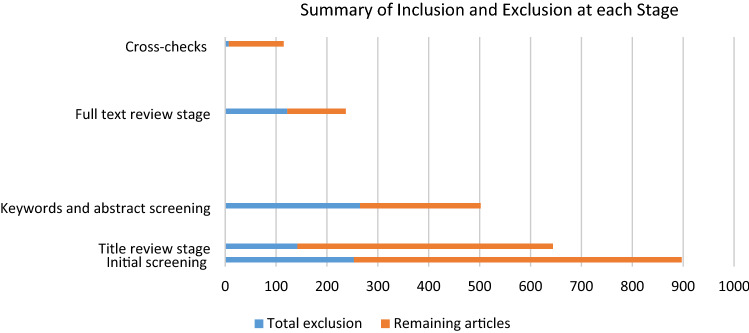
Summary of inclusion and exclusion at each stage

After reviewing the title, keywords, and abstract of the returned results, irrelevant articles were excluded and the rest of the articles were reviewed in their entirety, resulting in 108 articles shortlisted for the review. Table 1 shows the process of applying inclusion and exclusion criteria in detail and Fig. 2 summarizes Table 1. Figure 2 summarizes the total exclusion and remaining articles in each stage.

**Table 1:** Application of inclusion and exclusion criteria

Stages	Exclusion criteria	Number of articles excluded	Total exclusion	Remaining articles
Initial screening	Exclusion of duplicates	253	253	644
Title review stage	Non peer-reviewed articles	92	142	502
	Conference proceedings	37		
	Non-English	13		
Keywords and abstract screening	Articles focusing on technical engineering issues	43	265	237
	Articles on sustainable consumption and consumer behavior	64		
	Articles on sustainability in education and teaching and pure social sustainability	17		
	Articles on developed countries and economies	31		
	Articles focused on modular design and production	59		
	Articles on supply chain partners and alliances	31		
	Articles on flexible manufacturing optimization	20		
Full text review stage	Technical articles on life cycle assessment	28	122	115
	Articles on technology development for remanufacturing and recycling	43		
	Articles on city and urban transport	21		
	Articles on clean and renewable energies in supply chains	19		
	Articles on the development analytical tools for flexible supply chains	11		
Crosschecks	Articles lacking any implications for FGSCM strategies, practices, drivers, and barriers in emerging economy	07	07	108

Next, to extract the key themes covered in the shortlisted articles, the text-mining technique was employed. The finalized articles from the previous stage were imported into *NVivo12* for cross-validation. The articles were coded and categorized in terms of FGSCM conceptualization, operational impacts, strategies and practices, and drivers and barriers. All the authors were involved in coding and compiling the articles, which was later validated by an external researcher to ensure reproducibility of results and eliminating subjectivity. Text mining strengthened the validity and reliability of the selection process, including the finalized articles and the main themes. It also highlighted low values of relative frequencies as potential themes for future research.

Finally, to unearth the interconnection among the identified results, a network analysis was used. All major and minor categories and frequencies resulting from the previous stage were coded in a separate dataset and stored in *NVivo12* for network analysis. Conducting a network analysis on this dataset identified the knowledge gaps of FGSCM in emerging economies and revealed the studies with higher interconnection. A combined contingency and institutional theory lens were used to synthesize the findings and develop a research framework.

## Results of Systematic Literature Review

This section explains the stages of implementing the proposed research methodology and addressing the first research question on the status quo of FGSCM literature in emerging economies.

### Phase I: Initial Search in Academic Databases

**Fig. 3:**
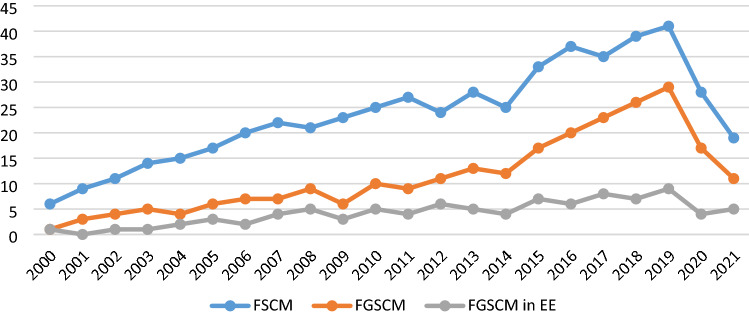
Comparative annual frequency of publications on general FGSCM and FGSCM in emerging economies

The concept of FGSCM gained its academic coverage in the 1990s (Fahimnia et al., 2015). However, most of the articles on FGSCM-related wider issues emerged after 2000 (Quarshie et al., 2016) followed by a sharp growth in academic publications afterward (Rafi-ul-Shan et al., 2018). Thus, the period used to conduct this review was determined to be from January 2000 to December 2021. Figures 3 compares the annual frequency of publications on general FGSCM and FGSCM in emerging economies indicating that, firstly, noticeably less research has been conducted on FGSCM in emerging economies and, secondly, the slope of increase is significantly lower for the latter.

### Phase II: Text Mining

**Fig. 4:**
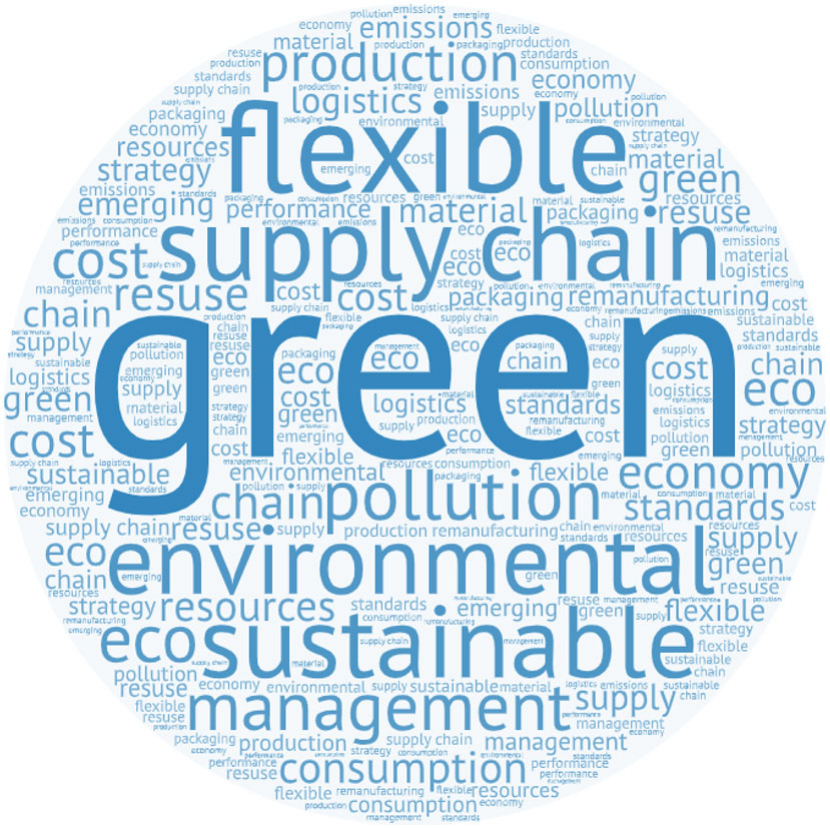
Word cloud of most frequently used words in the reviewed articles

Conducting text mining using *NVivo12* on the articles resulting from phase 1 facilitated visualization of the focus using word clouds as well as further analysis based on the industry sector, research methodology, and data analytics tools of the reviewed articles (Bin Makhashen et al., 2020). Figure 4 depicts the word cloud, highlighting the most frequently used words in the selected articles in bigger fonts, while other less frequent words appear in smaller fonts. A word cloud is a powerful visualization tool to identify common words in complex environments and facilitates unearthing dominant themes and keywords in a given context (Birko et al., 2015). The most frequently used words were “green” (word count: 5652), “supply chain” (4361), “sustainable” (4002), “environmental” (3794), “flexible” (3220), “management” (2431), and “emerging” (2187), followed by other keywords.

**Fig. 5:**
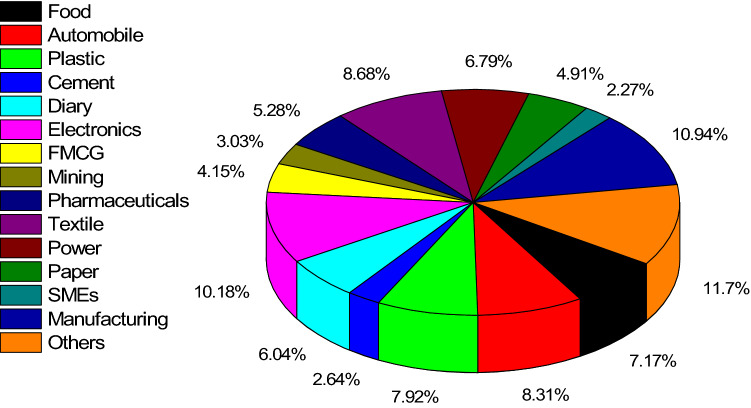
Analysis of articles by industrial sectors

The analysis of articles by industry sector suggests that the extant empirical research on FGSCM used various industrial sectors, as shown in Fig. 5. The top three industries were the manufacturing industry 11.2% (29 articles), electronics and electrical industries 10.42% (27 articles), and textile and apparel industry 8.88% (23 articles). A figure of 12% of reviewed articles (31 articles) did not disclose the industry. The reviewed articles were sorted based on the country on which the study focused. The results, shown in Table 2, reveal that India (43 articles) and China (38 articles) were by far the two highest-researched emerging economies.

**Table 2:** The frequency of emerging economies in the reviewed articles

No	Countries	Total number of publications	Percentage
1	India	43	18.00
2	China	38	15.90
3	Taiwan	27	11.30
4	Brazil	18	7.53
5	Bangladesh	17	7.11
6	Malaysia	15	6.27
7	South Africa	13	5.44
8	Mexico	09	3.76
9	Indonesia	07	2.93
10	Pakistan	05	2.10
11	Other countries	47	19.66

**Fig. 6:**
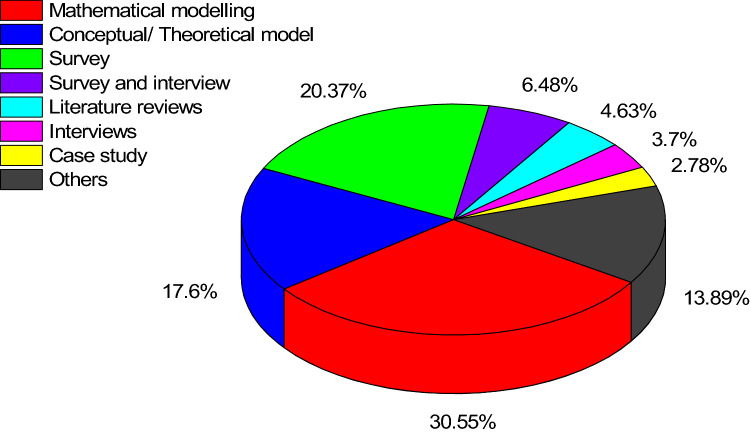
Analysis of articles based on research methodologies

**Fig. 7:**
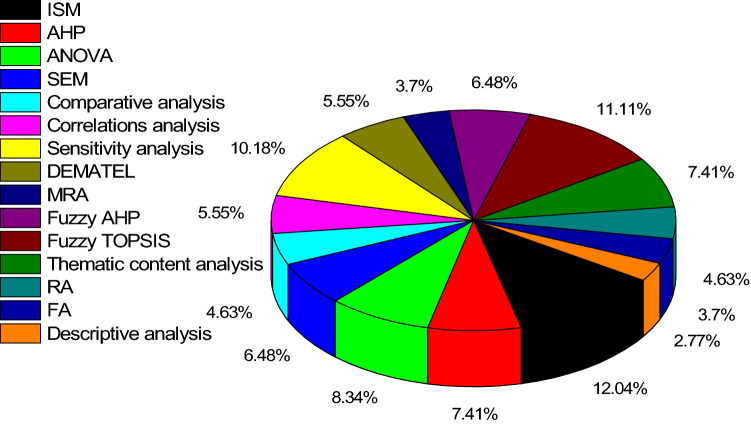
Analysis of articles based on application of analytics tools. AHP: Analytic Hierarchy Process, ANOVA: Analysis of Variance, DEMATEL: Decision making trial and evaluation laboratory, FA: Frequency Analysis, MRA: Multiple Regression Analysis, RA: Regression Analysis, SEM: Structural Equation Modeling

The articles were also analyzed based on research methodologies. As shown in Fig. 6, quantitative and mathematical modeling prevailed, which is at odds with the general trend of FGSCM literature. Our findings are at odds with Ansari and Kant (2017) who found more case studies and empirical qualitative studies, but are supported by Rajeev et al. (2017) who found that GSCM lacks qualitative research in the context of emerging economies when compared to the developed economies. It implies the maturity of literature on FGSCM as compared to FGSCM in emerging economies. Since quantitative methods were prevailing, the articles were further analyzed based on the applied data analytical tools, as shown in Fig. 7. Among various analytical tools applied, interpretive structural modeling (ISM) with 13 articles was the most popular data analysis technique, followed by Fuzzy TOPSIS (12 articles) and sensitivity analysis with 11 articles.

### Phase III: Network Analysis

**Fig. 8:**
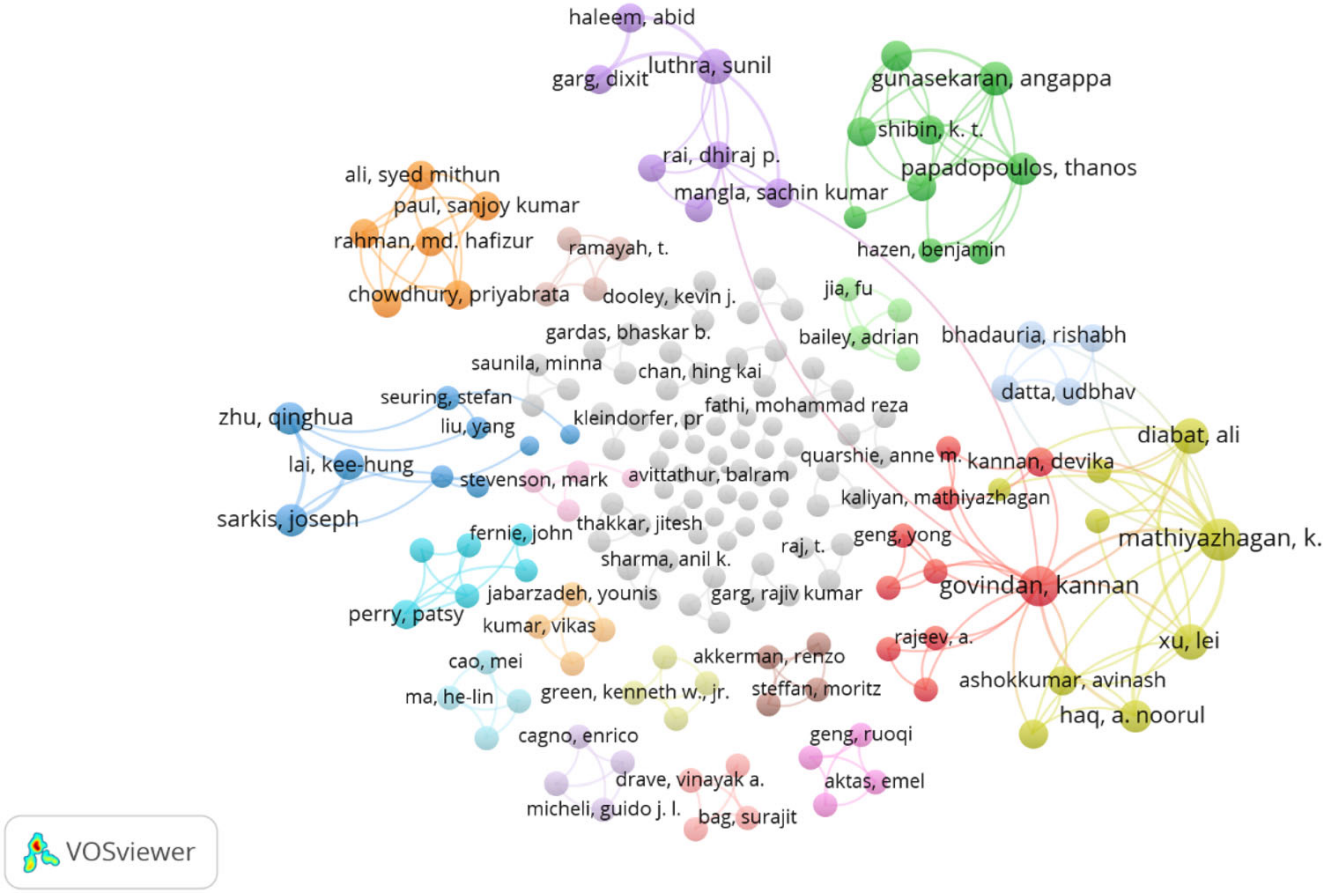
Network analysis of key articles

The articles with at least one citation were selected from the final list of articles, and their objectives and key findings were scrutinized to conduct network analysis. A network analysis was conducted on these articles. The results are depicted in Fig. 8.

It was found that FGSCM-related research is on the rise when compared to the FGSCM-related research in the context of emerging economies. Our network analysis demonstrated undirected empirical research on FGSCM in emerging economies, on the far edges of the network and distanced from the most cited FGSCM-related articles, enabling us to identify the most important contributions in the research domain. Based on the network analysis, key articles on FGSCM in emerging economies are highlighted. A summary of key contributions in the research domain is provided in the appendix.

## Thematic Analysis of the Literature

This section provides an in-depth analysis of the selected articles from the systematic literature review. Firstly, FGSCM strategies and practices were extracted. Building upon the contingency theory, they were juxtaposed with the general literature of FGSCM to identify the contextual specificities of emerging economies (second research question). Secondly, the drivers and barriers of FGSCM in emerging economies were identified and categorized based on the source of institutional pressure using the institutional theory (third research question).

### FGSCM Strategies and Practices

Many researchers attempted to identify and categorize FGSCM strategies and practices (e.g., Fang & Zhang, 2018; Nema et al., 2013; Srivastava, 2007; Zhu et al., 2007), but little research has been done on FGSCM strategies and practices in emerging economies and their specificities. Most of the terminologies, classifications, and categories of FGSCM strategies and practices were developed from the developed economies or network perspective. There are few studies that have researched the emerging economic context, such as those by Singh et al., (2020a, 2020b), Singh et al. (2019), and Singh and Acharya (2013) that extensively focused on developing a framework for supply chain flexibility. However, it was noticed that FGSCM strategies and practices received the least consideration in literature and practice. Thus, we identified FGSCM strategies and practices from the selected articles and juxtaposed them with the ones from the general FGSCM literature using the contingency theory lens. The results, summarized in Table 3, reveal that there are significant disparities between FGSCM strategies and practices in emerging economies and developed countries. These specificities should be considered when devising a FGSCM strategy or implementing practice in an emerging economy.

**Table 3:** FGSCM strategies and practices in emerging economies

FGSCM strategies	Related practices	Selected sources from emerging economies literature	Selected sources from general FGSCM literature	Specificities of emerging economies based on the contrasting selected sources
Green purchasing and supply management	Environmental audit for the internal management of suppliers Asking suppliers to acquire ISO 14001 certification Green supplier development programs	Vijayvargy et al. (2017) Diab et al. (2015) Liu et al. (2017) Luthra et al. (2016) Jabbour et al. (2020) Silvestre (2015) Adhikari and Bisi (2020)	Green et al. (2012) Touzi et al. (2015) Rao and Holt (2005) Zhu et al. (2008)	Corruption and distrust increase procurement costs and hinder green procurement in emerging economies Supplier–buyer relation is unregulated and involves higher informality in emerging economies Buying organizations from developed countries have higher bargaining power over the suppliers in emerging economies. Accordingly, they share less revenue with suppliers leading to lower greening quality
Green/flexible manufacturing and operations	Reusing, recycling and remanufacturing Environmental compliances, auditing programs, and total quality environmental management programs Flexible manufacturing and process development with environmental considerations	Govindan et al. (2015) Liu et al. (2017) Luthra et al. (2016) Jabbour et al. (2020) Adhikari and Bisi (2020) Raj et al. (2008)	Dubey et al. (2015) Srivastava (2007) Kleindorfer et al. (2005) Rafi-Ul-Shan et al. (2018)	In light of weaker regulations, internal stakeholders in emerging economies such as firm owners play a greater role in green / flexible manufacturing and operations than external stakeholders such as governments In emerging economies, the production is more labor-oriented rather than technology-oriented which leads to higher sustainability risks In emerging economies, green/flexible production is determined mostly when the cost–benefit analysis indicates profitability
Green logistic	Corporate environmental strategies toward logistics, reduction of carbon emissions and the use of a greener fleet Using greener modes of transport Using fuel with less carbon intensity Reduction of exhaust emission	Luthra et al. (2016) Lai and Wong (2012) Zhu and Sarkis (2016) Esfahbodi et al. (2016) Jakhar et al. (2018) Jawaad and Zafar (2019)	Cosimato and Troisi (2015) Kumar et al. (2015) Ali et al. (2016)	In emerging economies, sustainability terms are barely included in the contracts of third-party logistics providers Due to unaligned objectives of different organizational functions, green logistics would not necessarily lead to improved cost performance in emerging economies SMEs in emerging economies have just recently started considering green logistics initiatives such as minimizing empty miles and better space utilizations
Flexible reverse logistic and material recovery	Recovery of excess inventories and materials Sale of scrap, used materials and excess capital equipment Concurrent design of forward and reverse logistics Using the empty capacity of backhaul trucks for reverse logistics	Vijayvargy et al. (2017) Epoh and Mafini (2018) Fang and Zhang (2018) Jayaram and Avittathur (2015) Scavarda et al. (2019) Maric and Opazo-basaez (2019)	Thun and Müller (2010) Chiou et al. (2011) Gavronski et al. (2011) Gunasekaran and Spalanzani (2012) Zhu et al. (2013) Laosirihongthong et al. (2013)	Poor waste separation at the point of waste generation in emerging economies makes recovery and reverse logistics complicated Reverse logistics in emerging economies, especially in countries other than China, is still immature both from research and practice perspectives
Green / flexible product design and packaging	Selection of recyclable and biodegradable packaging materials Green product development by considering reuse, recycle, and recovery of materials, and the component parts at design stage	Liu et al. (2017) Luthra et al. (2016) Vijayvargy et al. (2017) Epoh and Mafini (2018) Zhu et al. (2008) Diab et al. (2015) Esfahbodi et al. (2016) Jayaram and Avittathur (2015) Geng et al., (2017a, 2017b)	Zhang and Zhao (2012) Chiou et al. (2011) Gavronski et al. (2011) Gunasekaran and Spalanzani (2012) Zhu et al. (2013) Gan et al. (2021)	In emerging economies, organizations adopt reactive strategies in acquiring green design standards such as ISO/TR 14,062 (e.g., when obliged by government or buyer), whereas in developed countries proactive strategies are prevalent In emerging economies, eco-design is justified mostly when it leads to profitability In emerging economies, governments’ investment is lower in waste management infrastructure and open-air landfilling is by far the most widespread method
Green marketing and customer relationship management	Use of environmentally friendly labeling of the products Encouraging customers to buy greener products Cooperation with customers for eco-design and greener packaging	Vijayvargy et al. (2017) Fang and Zhang (2018) Zhu and Sarkis (2016) Luthra et al. (2016) Geng et al., (2017a, 2017b) Jayaram and Scavarda et al. (2019) Jayaram and Avittathur (2015) Jawaad and Zafar (2019) Pakdeechoho and Sukhotu (2018)	Chan et al. (2012) Mobley et al. (1995) Essousi and Linton (2010) Wang et al. (2013) Scarpa and Willis (2010) Herring (2006) Young et al. (2010) Bai and Sarkis (2010) Nishitani (2010)	Due to lower awareness of customers in emerging economies about environmental issues, more information should be provided in product marketing as well as through product labels and leaflets In emerging economies, customer collaboration has a stronger impact on environmental performance, as compared to supplier collaboration In emerging economies, firms compete based on minimum environmental quality standards and low prices, rather than focusing on high environmental performance
Internal environmental management	Commitment of senior manager to FGSCM strategies Inter-departmental cooperation for environmental improvements Environment documentations and auditing	Zhu et al. (2008) Epoh and Mafini (2018) Vijayvargy et al. (2017) Luthra et al. (2016) Silvestre (2015)	Fang and Zhang (2018) Nema et al. (2013) Çankaya and Sezen (2019) Yu and Ramanathan (2015)	The firms in emerging economies poorly promote and implement employees’ environmental training and incentives. Environmental certifications such as ISO 14000 series are inadequate
Green/flexible innovation	Technologies for energy savings (Saunila et al., 2018) Using non-toxic raw materials Research and development orientation to green products	Zhang et al. (2019) Silvestre (2015) Pakdeechoho and Sukhotu (2018)	Abraham and Dao (2019) Saunila et al. (2018) Huang and Li (2017) Albort-Morant et al. (2016) Kumar et al. (2020)	Focal firms in emerging economies are the main determinants of green/flexible innovation. It is less likely that other supply chain members take initiatives Governments of emerging economies offer less sustainability incentives to motivate firms for innovation In emerging economies, green / flexible innovations are mostly concentrated at the end of product life, e.g., to repurpose the used product or its components, while in developed countries green/flexible innovation is placed throughout the supply chain including at the product design level
Green/flexible information system	Designing energy efficient IT equipment Reducing data centers energy consumption	Green et al. (2012) Abraham and Dao (2019) Silvestre (2015) Martín-Góme et al. (2019)	Gholami et al. (2013) Basaglia et al. (2009) Butler (2011) Elliot (2011) Gupta et al. (2019)	Inadequate infrastructures in emerging economies such as internet access, fiber optic, and information technology knowledge limit the implementation and efficiency of green information systems and negatively impact supply chain flexibility

### FGSCM Drivers and Barriers

Organizations in emerging economies face various drivers and barriers to adopt FGSCM strategies and practices. These pressures originate from different internal (from within the organization) and external (from outside the organization) stakeholders. This review unearthed major drivers and barriers and analyzed the sources from which they originated. Drivers related to government and regulations were categorized as coercive pressure. Drivers motivated by competitors or the cultural environment were considered as mimetic pressures. When a driver pertained to customer or market, it was perceived as normative pressure. Similarly, where a barrier was related to lack of government regulations or support, it was categorized as coercive pressure, indicating that governments should increase their support or exert further pressure to address the barrier. The same holds for barriers assigned to mimetic and normative pressures. When a driver or barrier originated from the internal environment of a firm, e.g., management commitment to environmental training, it is considered as an internal driver or barrier. For the sake of simplicity, when the analysis found *management support* as a driver and *lack of management support* as a barrier, for instance, it was only mentioned once in the list of drivers. Tables 4 and 5 provide a summary of identified drivers and barriers, respectively. These tables spotlight the two major key drivers/barriers (internal and external) of FGSCM in emerging economies.

**Table 4:** Key drivers of FGSCM in emerging economies

Key drivers	Source of institutional pressure	Description	Selected sources
External	Coercive	Government rules and regulations obliging firms to adopt FGSCM practices	Govindan et al. (2016)
			Mathiyazhagan et al.(2018)
			Mhelembe and Mafini (2019)
		Government incentives for funding, training and development of FGSCM initiatives	Pakdeechoho and Sukhotu (2018)
			Mangla et al. (2016)
			Govindan et al. (2016)
	Mimetic	Increased competition among organizations for green / flexible initiatives	Luthra et al., (2015a, 2015b)
			Mathiyazhagan and Haq (2013)
		Gaining competitive advantage over competitors by differentiation of products and services	Dhull and Narwal (2018)
			Gandhi et al. (2015)
		Competitors designing products with reusability, recyclability, or flexibility	Mathiyazhagan and Haq (2013) Mathiyazhagan et al. (2014)
		Competitors using cleaner technologies and renewable energies	Dhull and Narwal (2018)
			Dhull and Narwal (2016)
			Luthra et al., (2015a, 2015b)
		Adoption of FGSCM practices by suppliers and other supply chain partners	Gandhi et al. (2015)
			Bhool and Narwal (2013)
			Dhull and Narwal (2018)
			Gosling et al. (2010)
	Normative	Growing awareness among the customers for eco-friendly products	Bhool and Narwal (2013)
			Luthra et al., (2015a, 2015b)
		Improving organizational image in the eyes of customers	Bhool and Narwal (2013)
			Luthra et al., (2015a, 2015b)
		Better market for green products	Dhull and Narwal (2016)
			Gandhi et al. (2015)
Internal		Top management and employees’ willingness and commitment	Gandhi et al. (2015)
			Shibin et al. (2016)
		Organizational culture and policies	Mathiyazhagan and Haq (2013)
			Luthra et al., (2015a, 2015b)
			Shibin et al. (2016)
		Environmental training and development	Raut et al. (2017)
			Dhull and Narwal (2016)
		Organizations understanding the cost benefits of adopting FGSCM practices	Raut et al. (2017)
			Kumar et al. (2016)
			Shibin et al. (2016)

**Table 5:** Key barriers of FGSCM in emerging economies

Key drivers	Source of institutional pressure	Description	Selected sources
External	Coercive	Weak government regulations regarding FGSCM	Delmonico et al. (2018)
			Govindan et al. (2014)
			Sarker et al. (2021)
		Corruption and bribery	Silvestre (2015)
			Muduli et al. (2013)
		Intricate tax systems and overly bureaucratic government systems	Silvestre (2015)
			Muduli et al. (2013)
		Import–export regulations and transnational trade laws	Yadav et al. (2020)
			Govindan et al. (2014)
	Mimetic	Poor collaboration among supply chain partners to provoke FGSCM initiatives	Tumpa et al. (2019)
			Govindan et al. (2014)
			Shibin et al. (2016)
		Lack of suppliers’ and supply chain partners’ interest to implement FGSCM initiatives	Majumdar and Sinha (2019)
			Balaji et al. (2014)
			Shibin et al. (2016)
		Unavailability of third parties to collect used products	Balon et al. (2016)
			Govindan et al. (2014)
			Wang et al. (2015)
	Normative	Lack of customer interest or preference for FGSCM-related practices	Shohan et al. (2020)
			Govindan et al. (2014)
			Mangla et al. (2014)
		Lower customer demand for green/flexible products	Wang et al. (2015)
			Yadav et al. (2020)
		Weak promotion of green products	Tumpa et al. (2019)
			Lorek and Spangenberg, (2014)
			Khan and Qianli (2017)
Internal		FGSCM initiatives not included in organizational strategies and planning	Majumdar and Sinha (2019)
			Balon et al. (2016)
			Mangla et al. (2014)
		Unfit business models to support FGSCM	Govindan et al. (2014)
			Muduli et al. (2013)
		Resistance to change and unwillingness to adopt FGSCM practices	Delmonico et al. (2018)
			Dube and Gawande (2016)
		High costs of environment friendly products and/or green initiatives	Govindan et al. (2014)
			Balaji et al. (2014)
		Performance appraisal systems designed solely based on financial measures	Yadav et al. (2020)
			Govindan et al. (2014)
		Lack of appropriate technologies for green and flexible design, manufacturing, or recycling	Rahman et al. (2019)
			Mathiyazhagan et al. (2016)
			Mangla et al. (2014)
		Lack of information technologies systems for coordination and communication for FGSCM	Rahman et al. (2019)
			Majumdar and Sinha (2019)
		Lack of skilled workforce for FGSCM	Geng et al., (2017a, 2017b)
			Muduli et al. (2013)

## Toward a Research Framework for FGSCM in Emerging Economies

This section addresses the fourth research question about synthesizing the extant body of knowledge to inform future research on FGSCM in emerging economies. Firstly, it puts forward research gaps based on the findings of the systematic literature review. Next, it integrates the findings of the study and the research gaps to develop a research framework for FGSCM in emerging economies.

### Research Gaps to Inspire Future Research

Overall, the results confirm the findings of previous research (Geng et al., 2017a, 2017b; Silvestre, 2015) about the dearth of research on FGSCM in emerging economies. Despite the calls and dire need from a practice perspective, the research shows only a modest increase in this area. This section identifies the research gaps to develop a research framework and motivate future research.

#### Purchasing and Supply Management

The analysis of the word cloud reveals that some important themes in FGSCM such as purchasing and supply management are overlooked in emerging economies. Further analysis of institutional pressures shows that these areas are particularly important because larger multinational organizations often exert their purchasing power to increase their profit share, rather than to drive suppliers in emerging economies toward sustainability and flexibility. Moreover, no studies, apart from Adhikari and Bisi (2020), were found to investigate the effect of different types of contracts on sustainability.

***Gap 1: To further investigate green/flexible purchasing and supply management in emerging economies and particularly how supplier–buyer power imbalance influences mainstreaming sustainability and flexibility in supply chains.***

***Gap 2: To study the impact of contract terms and contract types, e.g., profit-sharing contracts or performance-based contracts on the sustainability and flexibility of firms in emerging economies.***

#### Industry and Country Analysis

The analysis of industries and countries show while some industries such as manufacturing, electronics, and the apparel industry received the most attention, other industries such as the service sector, SMEs, nonprofits, and development organizations are overlooked. This is an important observation as smaller firms in emerging economies are less equipped to develop FGSCM capabilities. Moreover, nonprofit and development organizations do not often account for sustainability in their strategy and operations (Zarei et al., 2019).

In terms of flexibility, our analysis of the literature shows that while the research has transcended beyond manufacturing flexibility in developed economies, as highlighted by Stevenson and Spring, (2007) and Yu et al. (2015), the interorganizational components of supply chain flexibility are still absent in emerging economies. In terms of countries, China and India received the greatest research attention while other emerging economies have been investigated less.

***Gap 3: To investigate FGSCM in the service sector, SMEs, nonprofit and development supply chains in emerging economies***

***Gap 4: To transcend research beyond firm level flexibility and account for interorganizational and supply chain flexibility in emerging economies***

***Gap 5: To study FGSCM in less explored emerging economies such as Mexico, Russia, South Africa, and Turkey and to conduct comparative cross-country analysis with the extant studies in China and India.***

#### Methodology and Theory

In terms of methodology, unlike the trend in general FGSCM literature where qualitative studies prevail (Ansari & Kant, 2017), the reviewed articles heavily used quantitative methods such as mathematical modeling (31%) and surveys (20%). Moreover, collaboration and action research aims at generating contextual knowledge (Coughlan & Coghlan, 2002), making it a perfect methodology to elaborate on the context of emerging economies. However, no participatory or action research methodologies were found during the review. In turn, the dearth of qualitative methods led to poor theory application and development. The review of Geng et al., (2017a, 2017b) identified that the majority of articles in emerging economies had not specified any theory in the period 1996–2015. Our review supports their findings and postulates that in the period 2000–2020, insufficient theory development, testing, and elaboration still prevails.

***Gap 6: To conduct case studies and participatory/action research and further theory application and development on FGSCM in emerging economies***

#### Context Specificity

Using the contingency theory lens helped to juxtapose the studies in general FGSCM with the ones in emerging economies (Table 3) and revealed that the contextual specificities in emerging economies reduce the slope of FGSCM evolution trajectory (supported by Silvestre, 2015). From the theoretical perspective, such specificities are the *contingency factors* in the context of an emerging economy that drive organizations to adopt different decisions vis-a-vis their operating context. Therefore, an interesting avenue for future research is studying how organizations, especially focal business firms, operating in emerging economies align their strategies and practices (response variables) to achieve a fit with these contingency factors (as context variables) to achieve more sustainable and flexible supply chains (as performance) (see: Sousa & Voss, 2008).

***Gap 7: To identify the specificities of emerging economies context, to explore how firms adjust their strategies and practices to cope with such specificities, and to assess the resulting flexibility and sustainability performance.***

Further comparison of the two contexts shows that in emerging economies, FGSCM strategies are often adopted only when they promise financial returns and are more likely to be abandoned if they fail in doing so (Esfahbodi et al., 2016), making proactive green strategy adoption less prevalent in emerging economies. Moreover, flexible reverse logistics, waste management, and green/flexible design were found to be the least developed strategies in emerging economies. The literature of FGSCM in developed countries can provide valuable insights for these areas.

As for other strategies, while present in both contexts, the implementation shows disparities. Customer collaboration in emerging economies leads to a stronger impact on environmental performance, as compared to supplier collaboration. Yet, studies on customer sustainability awareness in emerging economies are scarce. This motivates future research to explore strategies that improve customer collaboration and awareness in emerging economies. Furthermore, while research in developed countries indicates the positive impact of emerging technologies such as blockchain or industry 4.0 on FGSCM (Saberi et al., 2019), our review found no studies on the adoption of these technologies in emerging economies.

***Gap 8: To study how to customize or transfer benchmark strategies and practices of flexible reverse logistics, waste management, and green/flexible design from developed countries to emerging economies.***

***Gap 9: To identify the strategies that improve customer collaboration and awareness in emerging economies and evaluate their impact on the flexibility and sustainability performance of firms.***

***Gap 10: To investigate the impact of emerging technologies and concepts such as blockchain, industry 4.0, 3D printing, and big data on FGSCM in emerging economies***

#### Institutional Pressures

Identifying the sources of pressures on organizations that cause drivers or barriers on the path of FGSCM help managers to harness these pressures for mainstreaming FGSCM in emerging economies. The analysis presented in Tables 4 and 5 reveals more drivers and barriers related to the internal environment of firms, indicating that the (lack of) internal organizational support is an overriding (barrier) driver. This is in accordance with the findings of Jabbour et al. (2020) who expressed that company owners and shareholders are the most salient stakeholders to drive FGSCM in emerging economies. Little research, however, exists on the organizational functions and their impact on FGSCM in emerging economies.

***Gap 11: To investigate the internal organizational factors, from a functional perspective, that impact the adoption of FGSCM strategies and practices in emerging economies***

By taking an institutional theory lens, this review identified coercive pressures from government and regulations as powerful sources of compliance. However, fewer drivers and barriers related to coercive pressures were found, compared to the ones related to mimetic and normative pressures. This is notwithstanding the findings of Jayaram and Avittathur (2015) but is in line with Jabbour et al. (2020) about FGSCM-related coercive pressures in emerging economies.

Our findings suggests that business firms in emerging economies increasingly earn legitimacy by copying successful FGSCM strategies and practices of other firms (mimetic isomorphism) or due to customer and market pressures (normative isomorphism). It can imply that FGSCM in emerging economies is moving from mere compliance with regulations (coercive isomorphism) as firms are under increasing pressures by competitors and customers to gain legitimacy and market sustainability through FGSCM strategies and practices.

***Gap 12: To study the institutional pressures emanating from customers and competitors driving/impeding FGSCM strategies and practices in emerging economies***

### Developing a Research Framework of FGSCM in Emerging Economies

Developing research frameworks for flexible *or* sustainable SCM has been at the center of scholars’ attention as these frameworks synthesize and illustrate the status quo and future directions in a concise and visualized, yet inclusive, manner. Some examples of such research frameworks are, inter alia, Seuring and Müller (2008), Sarkis et al. (2011), Dubey et al. (2015), Rajeev et al. (2017), Liao (2020), and Carter et al. (2021). However, our survey of the literature shows the paucity of combined flexible *and* sustainable SCM frameworks in the context of emerging economies. Hitherto, extant research frameworks focused merely on one aspect of FGSCM in emerging economies such as barriers and enablers (see Delmonico et al., 2018; Rahman et al., 2019; Tumpa et al., 2019), or investigated the impact of FGSCM strategies and practices on environmental performance (e.g., Esfahbodi et al., 2016; Geng et al., 2017a, 2017b; Luthra & Mangla, 2018). The literature scants research frameworks that firstly delve into the specificities of emerging economies vis-à-vis combined flexible *and* sustainable SCM, and secondly inclusively synthesize strategies and practices, as well as drivers and barriers.

**Fig. 9:**
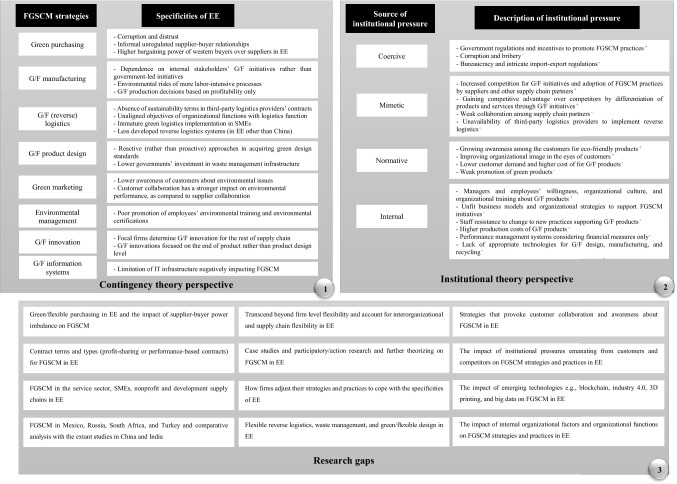
The proposed research framework for FGSCM in emerging economies

Our proposed research framework addresses these shortcomings. Firstly, it not only synthesizes FGSCM strategies and practices in emerging economies, but also it discerns the specificities of emerging economies using the contingency theory lens (Table 3). This helps managers and decision-makers to account for the contextual differences in emerging economies when devising their organizational strategies and practices. Secondly, the literature advocates that institutional pressures in emerging economies to adopt FGSCM strategies and practices are significantly different in emerging economies from developed countries (Raj et al., 2022). We have classified the identified drivers and barriers found from our systematic review, based on the source of pressure they originate using the institutional theory perspective (Tables 4 and 5). This classification deepens the understanding of policymakers about the institutional pressures in emerging economies and allows them to harness these pressures appropriately to promote FGSCM. Resulting from these observations, the research framework directs scholars to future research by identifying the main research gaps in the literature. The research framework is presented in Fig. 9.

## Conclusion

Emerging economies are prime targets of global businesses and multinational corporations for the outsourcing of manufacturing while the in-house operations that satisfy domestic demand in these countries are also seeing sharp growth (Jayaram & Avittathur, 2015). This paper reviewed the literature within the past 20 years and proposed a strategic research framework of FGSCM in emerging economies to address the increasing pressures from different stakeholders and calls from scholars to study FGSCM in emerging economies.

The study set out a systematic literature review to identify the status quo of research on the topic. The methodology is novel in that it combines a systematic literature review, text mining, and network analysis to explore, analyze, and synthesize knowledge gaps in the research domain. The applied inclusion and exclusion criteria, brainstorming sessions and crosschecks applied in consecutive steps contributed toward the selection quality of shortlisted articles and their subsequent analysis by limiting subjective biasness (Denyer & Tranfield, 2009). Text mining and network analysis of selected articles facilitated identifying networks of research articles dealing with particular aspects of FGSCM strategies and practices and showed that the extant empirical research articles in the research domain are fragmented.

Two grand organizational theories were employed: contingency theory to distinguish the specificities of emerging economies context, and institutional theory to discern the sources of pressures on institutions that facilitate or hinder FGSCM in emerging economies. Using contingency theory revealed that contextual specificities reduce the slope of FGSCM in emerging economies and using institutional theory revealed that coercive pressures from governments and regulations are powerful sources of compliances.

Finally, a research framework was developed to synthesize the extant literature and to identify the research gaps to inspire future research. This framework will help managers and decision-makers to understand the contextual differences in emerging economies while planning their organizational strategies and practices. Furthermore, the classification of drivers and barriers deepens the understanding of policy makers about the institutional pressures in the emerging economies allowing them to promote FGSCM.

This study is not devoid of limitations; firstly, the systematic literature review did not include studies with a mere focus on social sustainability. Higher prevalence of issues such as worker exploitation, unfair wages, substandard working environment, gender discrimination, and child labor in emerging economies are imperative avenues for future research. Secondly, the systematic literature review was restricted to the period of 2000–2020, to four academic databases, and to the research published in English. Some more important articles might exist outside our search boundaries.

Key Questions

1. What is the status quo of FGSCM research in emerging economies?
2. What are the specificities of FGSCM strategies and practices in emerging economies?
3. What are the sources of institutional pressure, i.e., drivers and barriers, to adopt FGSCM strategies and practices in emerging economies?
4. How can the extant body of knowledge inform future research on FGSCM in emerging economies?

## Appendix

See Table

**Table 6:** Analysis of key articles on FGSCM in emerging economies

Author(s)	Country	Objective of the research	Key Findings
Adhikari and Bisi (2020)	India	To explore collaboration mechanism using greening cost-sharing and profit-sharing contracts for FGSCM in apparel industry	It analyzes the bargaining process between the supply chain members for cost-sharing and profit-sharing contracts and investigates the impact of the fairness concern of the supply chain members on the greening and pricing decisions
Jawaad and Zafar (2020)	Pakistan	To study the impact of FGSCM on firm performance with a mediating role of investment recovery and competitiveness	FGSCM practices positive influence firm performance while investment recovery is a strong mediator between intra-organizational green activities and firm performance
Rahman et al. (2019)	Bangladesh	To identify and evaluate barriers of implementing FGSCM	Using a Fuzzy-VIKOR approach the following barriers were identified in the plastic industry of Bangladesh: inadequate knowledge and support, insufficient technology and infrastructure, financial constraints and unsupportive organizational and operational policies
Pinto et al. (2018)	BRICS	To explore and analyze the environmental management practices adopted by industries in BRICS countries in the period from 2011 to 2015	China, India and Brazil show a greater interest in disseminating research on environmental management issues whereas Russia and South Africa did not follow the same trend due to weaker environmental engagement
Delmonico et al. (2018)	Brazil	Identifying the barriers to sustainable procurement in Brazil	Five categories of barriers – organizational culture, motivation, economic uncertainty, market, and operations – were identified, out of which organizational culture is the most prominent one
Geng et al., (2017a, 2017b)	Asian emerging economies	To understand the relationship between FGSCM practices and firm performance in the manufacturing sector through a systematic literature review	According to the results of meta-analysis, FGSCM practices lead to better performance in economic, environmental, operational, and social performance. Other factors such as industry type, firm size, ISO certification, and export orientation are influential on the firm performance
Gopal and Thakkar (2016)	India	To analyze and identify critical success factors of sustainable supply management practices for Indian automobile industries	Sustainable supply chain performance (SSCP) was investigated in the Indian automobile industry. The result shows SSCP is correlated and helps in improving the supply chain performance among the industries surveyed. Moreover, environmental and social performance have a positive relationship with economic performance
Younis et al. (2016)	United Arab Emirates	To investigate the relationship between the implementation of FGSCM practices and their impact on corporate performance	It identified that FGSCM practices impacted the corporate performance differently and provided guidelines on what practice an organization should adopt considering the desired outcome
Chan et al. (2016)	China	To examine the mediating effect of green product innovation between the regulatory pressures and firm performance including the moderating effect of environmental dynamism on the relationship between green production innovation and firm performance	Environmental regulations have a positive impact on green product innovation whereas environmental dynamism has a relatively strong moderation effect on the relationship among green product innovation and cost efficiency
Jabbour and Jabbour (2016)	Brazil	To propose a synergistic and integrative framework for the green human resources management (GHRM) and FGSCM relationship and to propose a research agenda for this integration	It advocated GHRM-FGSCM integration from academic, managerial, and practical perspectives in the areas of organizational sustainability
Prasad et al. (2016)	India	To investigate the applicability of lean and green practices to foundry industries in India	It found that lean and green practices should be linked to enhance operational and environmental performance
Feng et al. (2016a, 2016b)	China	To examine how the relationship between environmental management systems and financial performance is moderated by switching cost, competitive intensity and their interaction	The results show a positive relationship between environmental management systems and financial performance. This relationship effects negatively by switching cost and effects positively by competitive intensity
Soundararajan and Brown (2016)	India	To examine the problems related to supplier facilities from a developing country supplier perspective	Due to suppliers' traditions, beliefs, local demands and resource dependency, codes of conducts fail in practice in emerging economies. In order to improve practices, buyer–supplier collaboration and trust is required
Chen et al. (2015)	China and India, and Sweden	To investigate the environmental management practices (EMPs) and firm performance in manufacturing companies of Sweden, China, and India	The results indicate that most EMPs do not have a positive correlation with financial performance, whereas a number of EMPs were found to have a strong correlation with improving innovation performance in various companies. Similarly, a negative correlation was found between the environmental standard for suppliers and sales growth
Jayaram and Avittathur (2015)	India	To analyze the expected changes to supply chain management by deploying FGSCM strategies	The article developed a conceptual grounded theory framework that linked environmental government policies to customer actions and firm sustainability strategies. It identified that green design, product recovery and reverse logistics are the key to FGSCM strategies
Park et al. (2015)	Indonesia	To analyze sustainability practices adopted by multinational companies in developing countries	This study found a critical need for a balance between centralized and decentralized governance for a successful sustainability strategy
Jia et al. (2015)	India	To analyze and identify the dominant SSCM practices in Indian mining and mineral industry	The article concluded that suppliers’ ISO14000 certification plays the most influential role over the recommended 25 SSCM practices. This certification is considered essential to increase sustainability performances in Indian mining and mineral industries
Lee (2015)	South Korea	To investigate the effects of FGSCM on environmental and operational performances considering social capital accumulation in the supply chain	The study found that FGSCM contributes to the environmental and operational performance improvements of the supply chain through social capital accumulation. Relational capital particularly plays a vital role in the relationships between FGSCM and environmental and operational performances
Luthra et al., (2015a, 2015b)	India	To identify, analyze and model the critical success factor to implement FGSCM toward sustainability in Indian mining industries	The article identified scarcity of natural resources as the most important critical success factor for FGSCM implementation and further developed a model to test its applicability in the Indian mining industry
Silvestre (2015)	Brazil	To study how supply chain sustainability can be implemented and managed in emerging economies through an evolutionary approach	This study found that the way a buyer company manager is influenced by its established network of relationships contributes to shaping the evolution of SSCM
Mansi (2015)	India	To explore the sustainable procurement practices across the Central Public Sector Enterprise in India	The study proposes a sustainable procurement index by analyzing the sustainability reports of 50 government owned companies in India. The study findings suggest a low adoption of green initiatives
Mathiyazhagan et al. (2015)	India	To investigate the pressures of FGSCM adoption and prioritize them	It identified pressures from non-governmental organizations for environmentally friendly products to motivate adoption of FGSCM in the Indian mining and mineral industry. Pressures from NGOs were ranked first, and financial factors were ranked last
Sen et al. (2015)	India and UK	To explore the role of environmental proactivity in financial performance in the context of manufacturing enterprises in India and the UK	The findings indicated positive correlation of environmental proactivity with financial performance and manufacturing and non-manufacturing based operational performance
Li et al. (2015)	Taiwan	To explore factors that impede implementation of environmental practices in eco-industrial parks	This study found that intellectual property rights restrict access to information and new technologies
Soda et al. (2015)	India	To investigate the level of adoption and implementation of FGSCM practices in an Indian context	The article focused on describing the adoption of FGSCM practices in India and identified operational efficiency as a primary driver. It further highlights the lack of literature on FGSCM in emerging economies
Ferreira et al. (2017)	Brazil	To examine the relationship between the maturity of environmental management and the adoption of FGSCM practices utilizing an integrative framework	Adherence to the integrative framework was verified, and sensitivity to changes in maturity of environmental management and the adoption of FGSCM practices were observed
Diabat et al. (2014)	India	To analyze the enablers for implementing FGSCM in Indian textile industries	This article identified five enablers for the implementation of FGSCM and reported that all the identified drivers were related to employee engagement
Ganapathy et al. (2014)	India	To identify the suitable combination of management and innovation practices that enables firms to eco-innovate and achieve sustainable performance	The role of management practice is more significant toward eco-innovation than innovative practices. Training on environmental related practices could tackle innovation and social factors in the Indian manufacturing sector context
Mitra and Datta (2014)	India	To explore the FGSCM practices and their impact on the performance in Indian manufacturing industries	This research identified green design, investment recovery and green purchasing practices that have a significant impact on the environmental performance of the firms
Mohanty and Prakash (2014)	India	To study whether the green score measures a common construct called FGSCM and whether internal and external pressures have any influence on FGSCM practices	The study investigated external factors such as suppliers, market pressure, competitors, and internal drivers such as on-the-job training and commitment of management, crucial for the implementation of FGSCM among the SMEs
Stiller and Gold (2014)	India	To explore the social SSCM practices in the Indian seed sector	It explored socially SSCM practices in the Indian seed sector. The research explored six types of practices, namely, reconceptualizing supply chain design, supply base continuity, decommunization, traditional supplier development, novel supplier development, transparency and traceability, and reward and incentive system
Zhu et al. (2013)	China	To investigate different types of institutional pressures motivating manufacturing enterprises to pursue FGSCM practices and commensurate performance outcomes	This study shows that institutional pressures have driven the manufacturer adoption of internal FGSCM practices which in turn relates to their external FGSCM practices adoption. The results from the statistical analysis also suggest that FGSCM practices indirectly improves economic performance
Jaikumar et al. (2013)	India	To identify the organizational factors that influence the environmental performance of companies	It identified factors such as pollution intensity, company size, collaboration with a foreign company and ISO 14000 certification to have a positive impact on the environmental performance of a company
Hsu et al. (2013)	Malaysia	To investigate the supply chain drivers that influence FGSCM initiatives in an emerging economy	The study found four important drivers of green supply chain adoption that collectively affect a firm’s green purchasing, design-for-environment and reverse logistics initiatives
Hultman et al. (2012)	Brazil and India	To investigate the firms’ responses to carbon markets in Brazil and India	There is no standard practice to account for financial benefits of clean development mechanisms investments. The non-financial reputational factors were the primary motivation for managers that are pursuing clean development mechanisms projects
Jayaraman et al. (2012)	India	To investigate the relationship between the consumer’s attitude toward the environment and the perceived image of a company that was environmentally conscious	Investigate relationship between the consumer’s attitude toward the environment and the perceived image of a company that was environmentally conscious. It found a strong correlation between the decision to buy a green product and the respondent’s concern for the environment as well as the perception formed of the firm
Koh et al. (2012)	India	To assess the adoption of low carbon and FGSCM practices by Indian service and manufacturing sectors	The research found that low carbon and FGSCM practice is a quality management initiative that requires bilateral efforts for the integration of the whole supply chain along with setting-up of supplier evaluation methods that ensures sustainable environmental operations
Zailani et al. (2012)	Malaysia	To investigate the extent of implementation of FGSCM practices and the outcomes of these practices on supply chain performance in Malaysia	The study found that FGSCM practices have a positive effect on sustainable supply chain performance and suggested that firms need to collaborate in advocating FGSCM practices as a route for firm’s commercial success rather than as a moral obligation
Rao et al. (2012)	India	To investigate if the environmental audit practices help the industries to increase productivity and maintain the environment at the same time	Identified various waste streams, waste minimization and treatment options in the distillery industry through environmental audit practice
Bhateja et al. (2011)	India	To identify the various FGSCM practices adopted both among SMEs and large-scale industries	Investigated factors that impact FGSCM integration and performance in the Indian manufacturing sector. Cost and lack of awareness about environmental issues were the main barriers but communication and collaboration for the integration of green initiatives was observed
Diabat and Govindan (2011)	India	To analyze different drivers affecting implementation of FGSCM using interpretive structural modeling	Analyzed drivers that affect implementation of FGSCM. It highlighted eleven drivers and an ISM model was developed and the interaction between the drivers were analyzed for the case study of the article
Munguia et al. (2010)	Mexico	To explore the pollution prevention practices performed by workers in the Mexican auto refinishing industry as well as their implications on the occupational, safety and environmental health of the workers and community	The findings indicate that the Mexican auto body shop industry is not consistent with the accepted precepts of sustainability. Mexican workers oftentimes perform their tasks under critical conditions which involves high level of pollution as well as considerable occupational and environmental risks
Zhu et al. (2010)	China	To examine whether different types of manufacturing enterprises on environmental-oriented supply chain cooperation exist and whether such cooperation impacts circular economy practices and economic and environmental performance outcomes in China	Findings suggest a need and importance to further strengthen the cooperation with upstream and downstream supply chain partners for a circular economy initiative to succeed
Rathore et al. (2011)	India	To understand the scenarios of remanufacturing and its implementation in the current socio-economic context	Investigated opportunities for establishing remanufacturing as a formal activity from the user perspective. A prescriptive model was proposed which utilizes the usage patterns of different consumer groups to create a self-sustainable demand–supply system
Park et al. (2010)	China	To analyze the integration of business values and environmental returns in the Chinese circular economy policy	Findings suggest that value creation within a supply chain can provide the impetus for organizations to adopt a circular economy and sustainable supply chain practices for competitive reasons. The four major business value dimensions include cost reduction, revenue generation, resiliency, and legitimacy and image
Sen (2009)	India	To investigate the relationship between the adoption of FGSCM practices and shareholder value creation	Investigated relationship between the adoption of FGSCM practices and shareholder value creation and found a positive correlation
Shukla et al. (2009)	India	To identify implementation level, major drivers, various practices and performance of environmentally and socially conscious SCM	Environmentally and socially responsive supply chains are in the early adoption stages in India. Research companies were aware of environmental and social issues, but actual implementation lacks a holistic approach
Zhu and Sarkis (2006)	China	To investigate the drivers and practices of FGSCM in Chinese manufacturing sector	The results suggest that FGSCM drivers are similar in the manufacturing sector, but FGSCM practices are industry specific
Luken and Stares (2005)	India, Pakistan, Sri Lanka, Thailand	To describe the findings of a project carried out in developing countries to help small and medium enterprises developing sustainable initiatives without compromising competitiveness	The results suggest that well targeted, enterprise-specific efforts to meet corporate social responsibility requirements can make a positive contribution to both short-term profitability and longer-term competitiveness
Zhu and Sarkis (2004)	China	To analyze the role of quality management and just-in-time practices as moderators in the adoption of FGSCM	Findings suggest that FGSCM practices tended to have win–win relationships in terms of environmental and economic performance. Quality management was found to be a positive moderator while JIT programs with internal environmental management practices may cause further degradation of environmental performance

6
